# Knowledge and attitude of organ donation in the Eastern region of Saudi Arabia and the influence of social media campaigns: a cross-sectional study

**DOI:** 10.1097/MS9.0000000000000258

**Published:** 2023-02-17

**Authors:** Mohammed Y. Alessa, Maryam S. Albedaiwi, Ali M. Al Mousa, Ghadeer M. Alhassan, Bayan T. Alnefaie

**Affiliations:** a King Faisal University; b King Faisal University, Hofuf, Saudi Arabia

**Keywords:** organ donation, organ transplantation, social media, Saudi Arabia

## Abstract

**Background:**

Previous studies showed organ donation to be less common in Arabic countries, and since there are not many studies exploring the perception toward organ donation, especially in the Eastern region of Saudi Arabia, this study aimed to assess the knowledge, attitude, and influence of social media campaigns on the willingness of organ donation.

**Methods:**

A cross-sectional study was conducted between September 2021 and April 2022 among social media users through an Arabic online survey exploring the population’s knowledge and attitude regarding organ donation and their willingness to donate. A total of 443 Saudi residents participated in the survey, with 376 of them meeting the required criteria.

**Results:**

In all, 85% of participants of the study’s calculated response rate were eligible to be included. One hundred ninety-eight of them (52.7%) were female, with age ranges between 18 and 29 years old (76.9%). The majority of the participants (63%) showed unwillingness to donate organs, with the highest rate among males (70.2%), aged above 30 (78.1%), and employees/students out of the healthcare sectors (69.5%). Inadequate knowledge was reported as the most common reason behind their rejection. Next to fears of operation and losing life, and the desire to donate to relatives only. Almost half of the participants (51.6%) agreed on the effect of social media on their behavior toward organ donation, with 187 of them (96.4%) referring to it as a positive effect which was significantly associated with the willingness to donate organs (*P*=0.006).

**Conclusion:**

The study results showed that most of the participants have neither proper knowledge nor willingness toward organ donation. Therefore, more strategies could be developed to increase the rate of donation in the Eastern region.

Highlights

- There is a huge gap between supply and demand in organ transplantation.
- The majority of study participants were unwilling to donate organs (63.03%), especially during their lifetime, with inadequate knowledge being the most reported cause.
- Social media applications seem to have a positive significant impact on organ donation willingness.

## Introduction

Organ donation is the donation of biological tissue or an organ of the human body to a recipient in need of transplantation^1^. It is the main preferable treatment for many end-stage organ diseases where no alternatives of comparable effectiveness exist. This type of treatment can make a huge transformation, saving and enhancing the quality of life of many patients^2^.

However, there is a huge gap between supply and demand in organ transplantation which forms an obstacle that resulted in increasing waiting lists in many countries around the world, including Saudi Arabia. Kidneys are the most needed organs in the Middle East, followed by the corneas, liver, and heart^3^. The total number of possible donors reported to the Saudi Center for Organ Transplantation (SCOT) between 1986 and 2019 was 13 731 possible donors. In 2019, only 585 deceased brain death donors were reported, and 113 of them were utilized^1^. Saudi Arabia has an organ donation rate estimated at 2–4 per million population, which is considered extremely low when compared to other Western and European countries, which reported a total of 18 147 deceased donors in the year 2019 only and a per million population ranging from 10 to 70 in some counties^4^,^5^.

In 1984, the SCOT was established to start adopting and developing strategies that included research conduction, distribution of donation cards, public awareness and health professionals’ education, and cooperating with charities to support organ failure patients^1^,^4^. The Eastern region was the second highest area to report donation after brainstem/brain death (DBD) donors and included the location of Dammam Medical Complex, which was the third top hospital contributing to the organ donation program in Saudi Arabia, and yet there were very few studies conducted to assess the knowledge and attitude in that area^1^. Most of the studies done in Saudi Arabia have shown very high awareness rates ranging from 70 to 90% and a satisfactory public enthusiasm of attitude regarding organ donation. Nevertheless, that promising response was not reflected in an increase in organ donation rates^4^,^6^–^8^.

According to Saudi General Authority for Statistics reports in 2019, Saudi youth (15–34) years old was forming (36.70%) of the total population, and the vast majority (98.43%) of them were using social media^9^. Many organ donation campaigns were recently launched in Saudi Arabia through social media to encourage the Saudi society to sign a card for organ donation and raise knowledge about organ donation and its impact on patients’ lives. The decision to actually donate organs is influenced by many factors, such as culture, knowledge, religion, and health status^7^–^10^. Understanding why people are not approaching to sign up for organ donation cards and whether the conducted strategies are effective or not is significant to build better-targeted campaigns and educational programs that will motivate more people to take action. Consequently, studies are required to assess the current situation and give a better insight into the regions that were not included in previous studies.

Therefore, assuming that social media campaigns have a noticeable positive effect on the population donating organs, this study aimed to assess the efficacy of social media campaigns on organ donation attitudes and to identify the causes of negative attitudes toward organ donation among the population of Eastern region of Saudi Arabia.

## Methods

This cross-sectional study was conducted from September 2021 to April 2022 by using a convenient sampling technique in the Eastern region of Saudi Arabia to assess the influence of social media on organ donation attitude and the associated factors that contributed to their decision. Using social media hits the target of seeking different age groups, and different sociodemographic statuses^7^, in addition to its efficacy and the huge campaigns that have been carried out recently. An online questionnaire was distributed on social media for 3 weeks in October 2021. In context to the study objectives, an Arabic survey was used to suit the general public, which has been tested many times to ensure its validity and reliability^7^. The included criteria were as follows: people who are living in the Eastern region in Saudi Arabia, between 18 and 60 years old, and who have heard of organ donation, to assess their knowledge. The exclusion criteria were as follows: people from areas other than the Eastern region, who are older than 60 years as it conflicts with the required criteria for organ donation, young children less than 18 years of age as underage are not mature enough to make an organ donation decision, and people who have not heard about organ donation previously, to eliminate possible false responses. Reducing the selection sampling bias was done by using diversity in distributing the survey on different social media applications and at multiple different times. The participants were also asked about being occupied in the health sector to determine potential confounding factors in the recruited sample.

The survey has been divided into five main sections: 12 questions regarding sociodemographic data; 11 questions to assess knowledge of organ donation and the attitude; 7 questions to explore the participant’s beliefs toward organ donation; 3 questions discussing the participant’s opinion of the beliefs behind the rejection of organ donation; and 2 questions to assess the effects of the social media on making the decision. The purpose of the study and the estimated time to answer the questions were provided with online consent, which was obtained prior to filling out the survey; however, all the participants who refused to give their consent were excluded.

A sample size formula for a single proportion was used to calculate the minimum sample size assuming that social media campaigns have positively influenced the public knowledge and attitude toward organ donation to be 57%, 95% confidence interval, and a sample error of 5%^11^. The questionnaire was filled out by 443 participants. After excluding participants who did not match the required criteria, the final sample size was 376, with an 84.9% response rate.

IBM SPSS Statistical Package for the Social Science, Windows 26.0 version was used to analyze the data, and Microsoft Excel 2016 was used to present the analyzed data on tables and graphs. The frequency of the questions was calculated to show the total responses for each choice the survey contains. As most of the questions were qualitative data, *χ* ^2^ was used as the main statistical analytical test to assess the knowledge, beliefs, and attitude toward organ donation. A *P* value of less than 0.05 was deemed significant.

The methods which have been used in this study were reported in line with Strengthening The Reporting Of Cohort Studies in Surgery (STROCSS) and Strengthening the Reporting of Observational Studies in Epidemiology (STROBE) guidelines^12^,^13^.

### Ethical consideration

The research complies with the ethical standards for conducting this study, as indicated by the scientific research committee at King Fahad Hospital, KFHH RCA NO: 34-43-2021.

## Results

A total of 443 responses have been received through an online survey. Three hundred seventy-six of them were eligible with the research criteria (response rate, 84.87%).

Table 1 demonstrates the sociodemographic characteristics of the participants, including gender, age, nationality, religion, educational level, employment, and marital status. Also, they have been asked whether they study/work in the healthcare sector or if they have a healthcare relative. All the previous questions have been presented in this table in relation to the willingness to donate an organ.

The average age of most of the participants was between 18 and 29 (76.86%), and they were almost evenly distributed between males (47.34%) and females (52.66%). The majority of the participants were unmarried, Saudi Muslim and undergraduate students. A total of 62.77% were not working in the healthcare sector. However, they do have a relative healthcare worker (55.59%). Most of the participants were not willing to donate organs, with a higher refusal rate shown in the male gender (70.22%), those above the age of 30 (78.16%), and divorced participants (83.33%).

To assess their beliefs toward organ donation, the participants were asked about the meaning of organ donation and their perception of it (Table 2). Also, their knowledge about organ donation requirements and their effect on both donor’s and recipient’s health have been assessed. The participants were also asked if a legal fatwa, an official pronouncement on legal issues with regard to Islam, had been issued allowing organ donation, nearly half of the participants answered yes (51.33%), and the other half ranged between no (2.13%) and I don’t know (46.54%). Another question about if there’s an influence of social media on their organ donation behavior and whether it is a positive or negative effect, 51.6% agreed on the social media effect, with 96.39% of them mentioning that it was a positive effect.

Table 3 shows the participants’ attitudes regarding organ donation. Participants were asked about their opinion on what prevents people from donating organs. Inadequate knowledge, fear of losing life, and fear of operation of 40.08%, 36.71%, and 35.86% showed the highest reported causes, respectively. In all, 81.91% of the participants preferred health education as a suggested method to increase the number of donors, whereas 60.63% suggested social media, 56.64% increased privileges for donors, and 38.56 used media, including television and daily newspapers.

However, 36.97% of the participants were willing to donate organs; 23.02% of them have a donation card already, 63.31% showed that they are willing to get one, whereas 13.67% refused to sign a donation card for several reasons, including the lack of family support, fears of complications after donation, and not having enough information about organ donation. The same group was asked about the preferred donation time, 58.27% chose to donate after death thinking it is easier after death and causes no problem, 6.47% chose during life because it is more humane, while others will be happier, and a very small number (5.17%) mentioned financial incentives. However, 35.25% showed the desire to donate both during life and after death.

**Figure 1:**
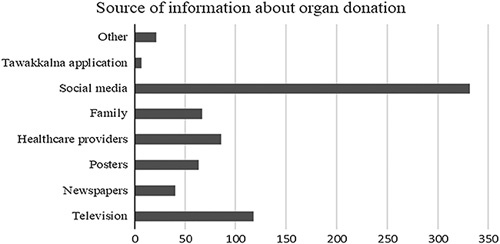
Source of information about organ donation.

Exploring the organs that can be donated (Table 3), the kidney was the most reported organ (78.5%), followed by the liver (67.1%) and heart (47.2%). Also, bone was the least organ known to be donated (4%). 47.07% of the participants think family consent is necessary even if the donor is brain dead and has an organ donation card. Relating to the research aim, a question regarding the source of information about organ donation was asked; the results are shown in Fig. 1 with social media as the most reported source (88.1%), television (31.3%), healthcare providers (22.8%), family (17.8%), posters (17%), newspapers (10.6%), and the least reported one was Tawakkalna application which is a national governmental platform that allows citizens to access many government services including signing for organ donation^14^.

When analyzing the concerned variables, some of them happened to have a significant relationship with the willing to donate organs, and some did not. Gender, age, and employment status all happened to have a significant relationship with a *P* value less than 0.05. Furthermore, the marital state showed an influence on the willingness to donate organs (*P*=0.0001). Besides being a student or employee in a healthcare sector (*P*=0.001), there was a significant relationship between willingness to donate organs and knowledge about organ donation requirements with the effect of organ donation on the recipient’s health (*P*=0.0001) (*P*=0.015), respectively. Also, religion seemed to have an influence on organ donation decision-making regarding an issued fatwa allowing donating organs in Saudi Arabia (*P*=0.001). Lastly, the social media programs’ impact showed a significant relationship with willingness to donate organs (*P*=0.0001) and the type of impact as well (*P*=0.006).

## Discussion

Organ donation campaigns appeared to be the focus of social media programs in recent years. Exploring the impact of these efforts on people’s knowledge and attitudes can help with the development of future initiatives to increase the number of organ donations among the Saudi population. Unfortunately, the majority of the participants were unwilling to donate organs (63.03%), especially during their lifetime, with inadequate knowledge being the most reported cause. The unwillingness to organ donation in this study can explain the huge gap between the demand and supply of organs^4^, although it was expected to have a difference in increasing the supply of donated organs in the current years as it has the advantage of easily obtainable information by social media. However, in terms of the study’s main goal, social media applications showed a significant relationship between the beliefs of the studied population toward organ donation and the willingness to donate organs. Surprisingly, neither education level nor having a healthcare worker as a relative had a significant impact on the willingness to donate organs (*P*=0.700) (*P*=0.786), respectively.

### Sociodemographic factors

Regarding the relationship between willingness to donate organs and the sociodemographic data, the study showed that there is a significant relation between age, gender, marital status, employment status, and belonging to the health field with a willingness to donate organs. In the same way, a previous study found that there was a significant correlation between age and educational level, but no relation was found with gender^2^. In contrast, another study conducted in Al-Kharj, found no significant relationship between the level of education and gender with the attitude toward donation; nevertheless, significance was reported with age^15^.

The study findings revealed a significant difference between males (70.22%) and females (56.57%) (*P*=0.006). This correlates to SCOT reports, where a gender analysis of brain-dead donors revealed that with a gender ratio of 3 : 1, 75% of DBD donors were men and 25% were women. Also, males made up 79% of potential DBD donors since 1986–2019, while females make up 21%, with a male-to-female ratio of 4:1^1^. Furthermore, participants who were aged 30 or more were more likely to be unwilling to donate organs. This is opposite to the actual DBD donors reported in Saudi Arabia. According to SCOT, an analysis of the age distribution of the potential DBD donors in 2019 revealed that 62% of the instances involved donors who were between the ages of 21 and 50, with the highest number in the age group between 41 and 50 years (22%). Furthermore, from the year 1986 up to 2019, 44% of instances involving potential DBD donors have been reported to be between the ages of 21 and 40^1^.

In respect of the observed unwillingness rate, a similar previous study done in Qatar showed that only 37.8% of Qataris and 32.8% of non-Qataris of its participants have the willingness to donate organs^16^. Moreover, a study in Hong Kong demonstrated that 43.3% of its participants were indecisive, while 25.4% refused the donation^17^. However, another research done in Saudi Arabia’s Western region found that only 13.2% of participants were unwilling to donate organs^9^. In addition, the majority of participants in other Arab countries were willing to donate their organs^18^,^19^.

The distinction between the results of this study population and other regions of Saudi Arabia and the Arab countries can be multifactorial. However, a study comparing rural and urban populations in Saudi Arabia found that rural areas have had a lower rate of willingness to organ donation than urban areas^20^. As the Eastern region incorporates a variety of geographical areas, many of which include a large rural population, in particular, Al-Ahsa governorate and Al-Qatif. The decrease in the willingness of people to donate organs in the results could be by virtue of increased rural participants in this study. In spite of this, a recent study done in the urban Jordanian population, reported that 68.2% and 78.2% of their participants were not willing to donate organs after death to save someone’s life and to improve scientific research, respectively^21^.

### Knowledge and attitude of organ donation

Most of the participants preferred to donate their organs after death, as it would not cause medical problems to them, the recipients would be happier as they would not see the donor suffering, more humanitarian and easier for the donor to do after death, even though 71.54% of the respondents were aware of the beneficence of organ donation on the recipient’s health. Furthermore, most of the participants believe in the necessity of family consent for organ donation if the donor is brain dead and has an organ donation card (47.07%). The necessity of family consent was explained in a previous study in 2014 as a lack of confidence in the healthcare workers’ reliability to establish a brain death diagnosis^22^, and an ongoing hope of the patient’s family of recovery. It is also important to note that according to SCOT’s 2019 annual report, the number of living organ donors was remarkably higher than deceased donation after brain death^1^, which is opposite to the subject’s attitude in the majority of national studies^7^,^15^,^20^. This could be explained by the tendency for people to donate to their relatives, which provides a motive to undertake living organ donation^23^.

According to previous studies, the reasons and factors contributing to organ donation can vary in different aspects, such as health status, legal, societal, traditional beliefs, and ethical boundaries^2^,^24^. In this study, many factors were expressed by the participants as a justification for their decision. 40.08% of unwilling participants did not have the required background and knowledge to encourage them to donate organs. Furthermore, fear of the operation or losing life have appeared to also be causes of refusing organ donation with a proportion of 35.86%, and 36.71%, respectively. A crucial role was played by the community knowledge, exhibited by the lack of information about the process and uncertainty of organ donation demonstrated in the sample’s response. Similarly, a lack of confidence in the healthcare system (31%), a fear of commercialism (27%), religious prohibition (19%), familial refusal (13%), and fear of surgery (10%) were reported in an Egyptian study to be associated factors^22^. There is a well-demonstrated relationship between religion and organ donation-related decisions in the literature. The majority of world religions, including Islam, support organ donation unless doing so constitutes harm to the donor’s life^25^,^26^. Furthermore, a Turkish study that included taking a class on religion, and the religious implications of organ donation showed that the provided class contributed to enhancing the study participants’ knowledge and altered their attitudes, making them more likely to become organ donors^27^. The health status of the recipient, relation to the recipient, religion of the recipient, and the age of the recipient were additional contributing factors on a previous study in the Kingdom of Saudi Arabia^2^.

Regarding knowledge about the requirements of organ donation, 175 (46.54%) of the participants answered ‘yes’ when asked about awareness of organ donation requirements, and 201 (53.46%) answered ‘no.’ Out of those who responded with yes, 26 subjects (14.86%) mentioned legal approval, 55 (31.43%) medical approval, 87 (49.71%) consent of the donor, and 42 (24%) psychological preparation of the donor. However, only 105 (60%), which accounts for less than one-third of the total participants, correctly responded with ‘all of the above’ (legal and medical approval, psychological preparation of the donor, and donor’s consent) as the answer for the requirements of organ donation.

The participants of this study had a relatively lower rate of knowledge of organ donation requirements when compared to a similar study done in Saudi Arabia, which was accompanied by a higher willingness rate (51%)^8^. In addition, significantly higher rates were found in a study including Qatari and non-Qatari populations, demonstrated as 52.5% and 63.5%, respectively^16^. The cultural composition of this study population could explain the diminished level of knowledge about the requirements of donation, as described in a preceding cultural study undertaken in Saudi Arabia^20^. Although no statistical significance was found between knowledge about the requirements and the willingness to donate organs in this study, the relationship between them was well defined in the literature as a dominant determinant in the attitude toward organ donation and to reduce the inadequacy of obtainable organs^25^. An Egyptian study assessed the acceptance of organ donation after death, which was undertaken through one-on-one counseling and a self-administrated questionnaire, illustrated a remarkable increase in posthumous organ donation acceptance, changing from 47% initially and reaching 78% after explaining the process of donation, its regulation and consenting form to the participants^28^. Thus, further addressing the importance of knowledge of organ donation requirements.

In respect of effect of organ donation on recipient’s health, the majority of responds believe that organ donation has a beneficial effect on the recipient’s health (71.54%), followed by 23.94% who are not aware of the donation effects, 2.93% believe there is no effect, and 1.6% believe donation is harmful on the recipient’s health. When compared to a preceding Saudi study, this study’s participants were more aware of the beneficial effects of organ donation on the recipient and had a lower proportion of participants who were not certain about the effects^7^. Likewise, Egyptian medical students, when asked about the benefit of organ donation to the recipient, 51 respondents (14.2%) were unsure, 18 respondents (5%) believed it was not useful, and 137 (38.2%) and 153 (42.6%) respondents thought it was beneficial or extremely beneficial, respectively^22^.

Regarding the knowledge of legal fatwa, this study, showed higher knowledge (51.33%) compared to a previous study that was done in Saudi Arabia in 2016 (46%)^7^. Even though nearly half of the participants have knowledge about the legal fatwa, most of them refused to donate their organs (55.4%). This might be due to inadequate knowledge, fears of operation, and fear of losing life^1^.

Exploring the organs that can be donated (Table 3), the kidney was the most reported organ to be known as a donatable organ in this study (78.72%), followed by the liver (67.29%), heart (47.34%), the cornea (40.96%), lungs (33.78%), pancreas (26.60%), and only 3.99% for bones. The high rate of kidney and liver as the most known organs to be donatable can be related to the fact that having two kidneys and donating one might not cause harm, in addition to the regrowing ability of the liver after cutting. Similarly, another study reported: the kidney (95.20%), followed by the liver (70.95%), heart (51.26%), eye (44.6%), and lung (43.6%)^15^. Contrary to this study, another study revealed that 85% of respondents were aware of eye donation, followed by the kidney (79.5%), heart (67%), liver (57%), lung (44.5%), skin (37.5%), pancreas (36%), bones (35%), and small intestine (32.5%)^29^.

### Effect of social media on decision-making

Regarding the source of information, similar to a previous study that was done in Saudi Arabia, the majority of participants received their information from social media networks and TV^8^,^20^ (Fig. 1). This suggests that the media has an impact on raising public awareness. Statistically, social media have a significant relationship with the decision to organ donation (*P*=0.0001).

Based on the influence of social media on their willingness to donate organs, more than half of the participants mentioned that social media had influenced their decision to donate organs in a positive way. At the same time, all of the individuals who stated that social media had a negative impact on their choice refused to donate their organs with a significant relationship (*P*=0.006). This demonstrates the importance of the media in raising organ donation awareness and influencing the practice of the population.

Moreover, the least reported source of information was the Tawakkalna application (1.9%), which is a national governmental platform that was created initially during the pandemic coronavirus disease 2019 to provide infection status along with organizing lockdown permissions and vaccine logistics. Afterward, it advanced to allow citizens to access many government services incorporating sectors of civil affairs, traffic services, government procedures, pilgrimage and Umrah services, educational services, and health-related services, including signing for organ donation^14^. Furthermore, the government arranged several campaigns to motivate and increase awareness about organ donation and its impact of it, such as Eithar, which is an authorized institution which has been developed as a whole program to increase organ donation through making public awareness campaigns, convincing the possible donors and their families about the importance of organ donation, beside the efforts that the Ministry of Health and The Saudi Center of Organ Transplantation are making through social media, posters, and schools campaigns.

## Conclusion

More than half of the studied subjects (63.03%) were unwilling to donate organs for a variety of factors that appeared to influence their decisions. Most importantly, having inadequate knowledge, fears of operations and loss of life, and the desire to donate to relatives only. These research findings strongly recommend increasing the work on future social media initiatives. Upcoming studies can further highlight the common and foremost strategies to implement social media in increasing the number of organ donors.

### Recommendations

The findings showed a positive relationship between social media and the perception regarding organ donation in the studied subjects, which could be further used to increase the willingness by initiating more focused campaigns. Also, family consent is necessary, especially in brain-dead patients and after death, even with the existence of donation cards which unfortunately showed high rejection. A step should be taken to correct the families’ misbeliefs, and more education is required. Another option is to consider the donation card as sufficient consent to donate without referring to family. Lastly, incentives, including financial incentives could contribute to increasing the rate of donation.

### Limitations

As a limitation of this study, the data was collected using an online survey, an issue in which only people who use social media platforms on a regular basis have a chance to participate. Furthermore, this study included a modest number of participants and was primarily filled by university students. So, we expect future researchers to do this study on a larger population, including all subgroups of the region in order to get results that can be generalized.

## Ethical Approval

The research complies with the ethical standards for conducting this study, as indicated by the scientific research committee at King Fahad Hospital, KFHH RCA NO: 34-43-2021.

## Sources of funding

This study is supported by the Deanship of Scientific Research, Vice Presidency for Graduate Studies and Scientific Research, King Faisal University, Saudi Arabia, Project No. 1986.

## Author contribution

M.Y.A.: mentor; M.S.A., A.M.A.M., G.M.A., and B.T.A.: study design, data collection, data analysis, and writing.

## Conflicts of interest disclosure

The authors declare no conflicts of interest.

## Research registration unique identifying number (UIN)

1. Name of the registry: none (clarified below).
2. Unique identifying number or registration ID: none (clarified below).
3. Hyperlink to your specific registration (must be publicly accessible and will be checked): none (clarified below).

## Clarification

As per the International Journal of Surgery policies, this study is a cross-sectional survey-based study and didn’t include neither patients nor human experiments or involvement of any kind. The study targeted the public community of Eastern region of Saudi Arabia. Therefore, does not include or require a research registration unique identifying number (UIN) or registration ID. As the above-mentioned registries target clinical trials, our research does not meet their criteria.

## Guarantor

Dr Mohammed Yousef Alessa.

## Provenance and peer review

Not commissioned, externally peer-reviewed.

**Table 1:** The sociodemographic characteristics of the participants in relation to willingness to organ donation (*n*=376) (37% willing to donate organs) (63% unwilling to donate organs)

		Are you willing to donate organs?		
**Item**	*N* (%)	Yes, *N* (%)	No, *N* (%)	*P*
Gender*				0.006
Male	178 (47.34)	53 (29.78)	125 (70.22)	
Female	198 (52.66)	86 (43.43)	112 (56.57)	
Age*				0.001
18–29	289 (76.86	120 (41.52)	169 (58.48)	
Above 30	87 (23.14)	19 (21.84)	68 (78.16)	
Nationality				0.888
Saudi	371 (98.67)	137 (36.93)	234 (63.07)	
Non-Saudi	5 (1.33)	2 (40)	3 (60)	
Religion				0.896
Muslim	373 (99.2)	138 (37)	235 (63)	
Non-Muslim	3 (0.8)	1 (33.33)	2 (66.67)	
Educational level				0.700
Primary school	1 (0.27)	0 (0)	1 (100)	
Middle school	5 (1.33)	2 (40)	3 (60)	
High school	76 (20.21)	26 (34.21)	50 (65.79)	
Undergraduate	285 (75.8)	106 (37.19)	179 (62.81)	
Postgraduate	9 (2.39)	5 (55.56)	4 (44.44)	
Employment*				0.043
Employed	84 (22.34)	23 (27.38)	61 (72.62)	
Unemployed	37 (9.84)	12 (32.43)	25 (67.57)	
Student	251 (66.76)	104 (41.43)	147 (58.57)	
Retired	4 (1.06)	0 (0)	4 (100)	
Marital state*				0.000
Unmarried	254 (67.55)	112 (44.09)	142 (55.91)	
Married	116 (30.85)	26 (22.41)	90 (77.59)	
Divorced	6 (1.6)	1 (16.67)	5 (83.33)	
Health sector employee/student*				0.001
Yes	140 (37.23)	67 (47.86)	73 (52.14)	
No	236 (62.77)	72 (30.51)	164 (69.49)	
Having a healthcare relative				0.786
Yes	209 (55.59)	76 (36.36)	133 (63.64)	
No	167 (44.41)	63 (37.72)	104 (62.28)	

* Significant (*P*<0.05).

**Table 2:** The relationship between the beliefs toward organ donation and the willingness to organ donation (*n*=376) (37% willing to donate organs) (63% unwilling to donate organs)

		Are you willing to donate organs?		
Item	*N* (%)	Yes, *N* (%)	No, *N* (%)	*P*
The term ‘organ donation’ means				0.122
Remove a human organ from a corpse	46 (12.23)	10 (2.66)	36 (9.57)	
Removing a human organ from a living person	4 (1.06)	1 (0.27)	3 (0.8)	
Removing an organ from one human being to another human being for the purpose of replacing the injured or nonexistent organ	92 (24.47)	38 (10.11)	54 (14.36)	
All the above	234 (62.23)	90 (23.94)	144 (38.3)	
Perception of organ donation^a^				0.062
To save someone’s life	369 (98.14)	137 (36.44)	232 (61.7)	
Out of compassion/sympathy	127 (33.78)	54 (14.36)	73 (19.41)	
For money	54 (14.36)	18 (4.79)	66.7 (17.74)	
As a responsibility	51 (13.56)	24 (6.38)	27 (7.18)	
Other	10 (2.66)	7 (1.86)	3 (0.8)	
Knowledge about requirements of organ donation*				0.000
Yes	175 (46.54)	82 (21.81)	93 (24.73)	
No	201 (53.46)	57 (15.16)	144 (38.3)	
If yes, what are they? (*n*=175)^a^				0.323
Legal	26 (14.86)	11 (6.29)	15 (8.57)	
Medical	55 (31.43)	20 (11.43)	35 (20)	
Consent of the donor	87 (49.71)	38 (21.71)	49 (28)	
Psychological preparation of the donors	42 (24)	15 (8.57)	27 (15.43)	
All the above	105 (60)	52 (29.71)	53 (30.29)	
Effect of organ donation on donor’s health				0.736
No effect	76 (20.21)	30 (7.98)	46 (12.23)	
Harmful effect	50 (13.3)	20 (5.32)	30 (7.98)	
I don’t know	222 (59.04)	81 (21.54)	141 (37.5)	
Beneficial effect	28 (7.45)	8 (2.13)	20 (5.32)	
Effect of organ donation on recipient’s health*				0.015
No effect	11 (2.93)	1 (0.27)	10 (2.66)	
Harmful effect	6 (1.6)	1 (0.27)	5 (1.33)	
I don’t know	90 (23.94)	25 (6.65)	65 (17.29)	
Beneficial effect	269 (71.54)	112 (29.79)	157 (41.76)	
Has a legal fatwa been issued allowing organ donation in the Kingdom of Saudi Arabia?*				0.001
Yes	193 (51.33)	86 (22.87)	107 (28.46)	
No	8 (2.13)	5 (1.33)	3 (0.8)	
I don’t know	175 (46.54)	48 (12.77)	127 (33.78)	
Have social media programs contributed to your behavior toward organ donation?*				0.000
Yes	194 (51.6)	98 (26.06)	96 (25.53)	
No	182 (48.4)	41 (10.9)	141 (37.5)	
If the answer is ‘yes,’ was the impact of social media programs positive or negative?*				0.006
Positive effect	187 (96.39)	98 (50.52)	89 (45.88)	
Negative effect	7 (3.61)	0 (0)	7 (1.86)	

* Significant (*P*<0.05).

^a^ Multiple answers are possible.

**Table 3:** The participants’ attitude regarding organ donation (*n*=376)

Item	*N* (%)
Are you willing to donate organs? (*n*=376)	
Yes	139 (36.97)
No	237 (63.03)
If no, why? (*n*=237)^a^	
Inadequate knowledge	88 (37.13)
Afraid of operation	66 (27.85)
Fear of losing life	50 (21.10)
Desire to donate to relatives only	42 (17.72)
Medical reason	28 (11.81)
All the above	7 (2.95)
I don’t know	25 (10.55)
Other	70 (29.54)
In your opinion, what causes people not to donate organs ‘if the answer is no?’ (*n*=237)^a^	
Inadequate knowledge	95 (40.08)
Afraid of operation	85 (35.86)
Fear of losing life	87 (36.71)
Desire to donate to relatives only	32 (13.50)
All the above	79 (33.33)
I don’t know	34 (14.35)
In your opinion, what causes people not to donate organs ‘if the answer is yes?’ (*n*=139)^a^	
Inadequate knowledge	56 (40.29)
Afraid of operation	58 (41.73)
Fear of losing life	53 (38.13)
Desire to donate to relatives only	14 (10.07)
All the above	65 (46.76)
I don’t know	10 (7.19)
What do you think of the methods to increase the number of donors ‘if the answer is no?’ (*n*=237)^a^	
Health education	183 (77.22)
Privileges for donors	132 (55.70)
Media (television/daily newspaper)	80 (33.76)
Social media	124 (52.32)
I don’t know	26 (10.97)
What do you think of the methods to increase the number of donors ‘if the answer is yes?’ (*n*=139)^a^	
Health education	125 (89.93)
Privileges for donors	81 (58.27)
Media (television/daily newspaper)	65 (46.76)
Social media	104 (74.82)
I don’t know	4 (2.88)
If yes, when? (*n*=139)	
During life	9 (6.47)
After death	81 (58.27)
Both	49 (35.25)
If the answer is ‘during life,’ why? (*n*=58)^a^	
More humane	41 (70.69)
Cause no problem	12 (20.69)
Others will be happier	33 (56.90)
Organ donation is easier during life	9 (15.52)
Financial incentives	3 (5.17)
What organs will you donate during your life? (*n*=58)^a^	
Kidneys	53 (91.38)
Bone marrow	28 (48.28)
Liver	39 (67.24)
Bone	6 (10.34)
If the answer ‘after death,’ why? (*n*=130)^a^	
More humane	56 (43.08)
Cause no problem	84 (64.62)
Others will be happier	67 (51.54)
Organ donation is easier after death	88 (67.69)
What organs will you donate after death? (*n*=130)^a^	
Kidneys	115 (88.46)
Bone marrow	96 (73.85)
Liver	113 (86.92)
Bone	77 (59.23)
Heart	107 (82.31)
Lungs	100 (76.92)
Cornea	87 (66.92)
Pancreas	91 (70.00)
Who are you willing to donate to? (*n*=139)^a^	
Parents	121 (87.05)
Sons and daughters	117 (84.17)
Friends	114 (82.01)
Relatives	115 (82.73)
Non-relatives	114 (82.01)
What organs can be donated: (*n*=376)^a^	
Kidneys	296 (78.72)
Heart	178 (47.34)
Lungs	127 (33.78)
Liver	253 (67.29)
Cornea	154 (40.96)
Pancreas	100 (26.60)
Bone	15 (3.99)
I don’t know	82 (21.81)
Are you willing to sign a donation card? (*n*=139)	
Yes	88 (63.31)
No	19 (13.67)
Already have one	32 (23.02)
If no, why? (*n*=19)^a^	
Worries about receiving inadequate health care after donation	11 (57.89)
Lack of family support	11 (57.89)
Lack of incentives	3 (15.79)
Don’t have enough information about organ donation	15 (78.95)
Fear of complications after organ donation	11 (57.89)
Religious reasons	6 (31.58)
Other reasons	9 (47.37)
Is family consent necessary even if the donor is brain dead and has an organ donation card?	
Yes	177 (47.07)
No	88 (23.40)
I don’t know	111 (29.52)

^a^ Multiple answers are possible.
